# How Low Can You Go: Methane Production of *Methanobacterium congolense* at Low CO_2_ Concentrations

**DOI:** 10.3389/fbioe.2019.00034

**Published:** 2019-03-07

**Authors:** Xihan Chen, Lars Ditlev Mørck Ottosen, Michael Vedel Wegener Kofoed

**Affiliations:** Section for Biological and Chemical Engineering, Department of Engineering, Aarhus University, Aarhus, Denmark

**Keywords:** methanogenesis, hydrogen, carbon dioxide, biomethanation, CO_2_ threshold, CO_2_ kinetics, biogas upgrading

## Abstract

Autotrophic hydrogenotrophic methanogens use H_2_/CO_2_ as sole carbon and energy source. In contrast to H_2_, CO_2_ is present in high concentrations in environments dominated by methanogens e.g., anaerobic digesters (AD), and is therefore rarely considered to be a limiting factor. Nonetheless, potential CO_2_ limitation can be relevant in the process of biomethanation, a power-to-gas technology, where biogas is upgraded by the addition of H_2_ and ideally reduce the CO_2_ concentration in the produced biogas to 0–6%. H_2_ is effectively utilized by methanogens even at very low concentrations, but little is known about the impact of low CO_2_ concentrations on methanogenic activity. In this study, CO_2_ consumption and CH_4_ production kinetics under low CO_2_ concentrations were studied, using a hydrogenotrophic methanogen, *Methanobacterium congolense*, as model organism. We found that both cellular growth and methane production were limited at low CO_2_ concentrations (here expressed as Dissolved Inorganic Carbon, DIC). Maximum rates (*V*_max_) were reached at [DIC] of 100 mM (extrapolated), with a CO_2_ consumption rate of 69.2 *f*mol cell^−1^ d^−1^ and a CH_4_ production rate of 48.8 *f*mol cell^−1^ d^−1^. In our experimental setup, 80% of *V*_*max*_ was achieved at [DIC] >9 mM. DIC half-saturation concentrations (*K*_m_) was about 2.5 mM for CO_2_ consumption and 2.2 mM for CH_4_ production. No CH_4_ production could be detected below 44.4 μM [DIC]. These data revealed that the limiting concentration of DIC may be much higher than that of H_2_ for a hydrogenotrophic methanogen. However, DIC is not a limiting factor in ADs running under standard operating conditions. For biomethanation, the results are applicable for both *in situ* and *ex situ* biomethanation reactors and show that biogas can be upgraded to concentrations of 2% CO_2_ (98% CH_4_) while still retaining 80% *V*_*max*_ at pH 7.5 evaluated from *M. congolense*. Since DIC concentration can vary significantly with pH and *p*CO_2_ during biomethanation, monitoring DIC concentration through pH and *p*CO_2_ is therefore important for keeping optimal operational conditions for the biomethanation process.

## Introduction

Methanogenic archaea play a key role in the production of biogas from anaerobic digesters (AD), yielding a product gas with 50–75% CH_4_ and 25–50% CO_2_ (Plugge, 2017). Methanogenic archaea here produce CH_4_ from either H_2_/CO_2_ (hydrogenotrophic methanogensis, 4H_2_ + CO_2_ → CH_4_ + 2H_2_O) or acetate (acetoclastic methanogensis, CH_3_COOH → CH_4_ + CO_2_). Hydrogenotrophic methanogens are ubiquitous in natural anaerobic environments other than engineered AD systems, e.g., the gastrointestinal tracts, flooded soils, and anoxic lake and marine sediments (Whitman et al., 2014). In anaerobic environments, H_2_ is an intermediate produced by fermentative and syntrophic bacteria, where it undergoes rapid turnover and its concentration is extremely low (Lin et al., 2012). Use of H_2_ as an electron donor is however not restricted to hydrogenotrophic methanogens, but other anaerobic microorganisms, e.g., sulfate reducers and acetogens compete for available H_2_ with methanogens in anoxic environments (Robinson and Tiedje, 1984; Cordruwisch et al., 1988; Kotsyurbenko et al., 2001). Therefore, many studies have been committed to the understanding of H_2_ uptake kinetics of hydrogenotrophic methanogens through either pure cultures or the whole microbial community in environmental samples (e.g., Conrad, 1999; Kotsyurbenko et al., 2001; Eecke et al., 2012, 2013).

In an AD, dissolved H_2_ concentration is usually low [0.5–3 μM, (Frigon and Guiot, 1995)]. Low H_2_ concentration limits methane production through hydrogenotrophic methanogenesis, which has been verified by many studies devoted to biogas upgrading by injecting H_2_ directly into the AD (Luo and Angelidaki, 2012; Agneessens et al., 2017). Above studies showed that the addition of H_2_ to ADs greatly increases methane concentration in the biogas, while decreasing CO_2_ concentration—a process known as biomethanation.

Through biomethanation the CH_4_ concentration is increased to as high as natural gas quality (>95%), and thus this process dramatically alters the standard operational conditions in AD because CO_2_ concentration is correspondingly reduced to lower than 5%. Such low CO_2_ concentration is rarely seen in natural anaerobic environments where methanogens are present, so it is not clear whether such low CO_2_ concentration affects the activities of hydrogenotrophic methanogens. However, CO_2_ is known to be an important substrate for hydrogenotrophic methanogens, as it serves as both electron acceptor for energy production and (sole) carbon source for biosynthesis through the Wood-Ljungdahl pathway (Berg, 2011; Borrel et al., 2016). To the best of our knowledge, there is limited knowledge about CO_2_ uptake kinetics of methanogens in literature. Nevertheless, understanding CO_2_ uptake kinetics of methanogens could consequently be crucial when dealing with the concept of biomethanation, which aims at upgrading the CH_4_ concentrations in biogas to >95% by consuming CO_2_ to as low concentration as possible.

Previous work gives some insights about limitation of CO_2_ consumption rate and methanogenic rate at low CO_2_ concentrations during biomethanation (Luo et al., 2012; Garcia-Robledo et al., 2016; Agneessens et al., 2017). Here it was shown that H_2_ consumptions rate decreased when headspace CO_2_ concentrations was lower than 12% during H_2_ pulse injection batch experiments in bioreactors (Agneessens et al., 2017), while an inhibition of H_2_ consumption rate was found when CO_2_ concentration was below 6% in a methanogenic manure samples (Garcia-Robledo et al., 2016). These results thus indicate that CO_2_ uptake rate and methanogenic rate is limited at low CO_2_ concentrations with great implications for the limits of the biomethanation technology. However, the exact impact of CO_2_ on the methanogenic activity was not clearly depicted by those studies, as they were conducted on complex microbial communities that include both CO_2_ consumers and producers—including homoacetogens that compete with methanogens for H_2_ and CO_2_. Therefore, a thorough understanding of methanogenic reaction kinetics at low CO_2_ concentrations seems necessary in order to find out under which conditions hydrogenotrophic methanogenic rates will be reduced or even inhibited during biomethanation. This will enable us to optimize the efficiency of biomethanation.

In this study, we present the first trial to examine CO_2_ uptake kinetics of a hydrogenotrophic methanogen, *Methanobacterium congolense*, by studying its CH_4_ production and CO_2_ consumption rates at low CO_2_ concentrations with surplus of H_2_. We chose to study a model organism from the genus *Methanobacterium* because methanogens of this genus were found to increase substantially after pulse H_2_ injections in reactors with mesophilic sludge (Agneessens et al., 2017). *M. congolense* was used as test strain because it is a mesophilic methanogen originally isolated from a mesophilic anaerobic digester, and also because it solely utilizes H_2_ and CO_2_ as substrates for growth and methane production (Cuzin et al., 2001).

## Materials and Methods

### Strains and Culture Medium

Type strain *Methanobacterium congolense* (DSM7095) was purchased from Leibniz-Institut DSMZ-Deutsche Sammlung von Mikroorganismen und Zellkulturen GmbH (DSMZ). The methanogen was cultivated with a H_2_:CO_2_ (4:1) gas mixture at 37°C in versatile medium for methanogenic archaea, based on the medium developed by Khelaifia et al. (2013), but adding only acetate, formate and NaHCO_3_ as carbon source.

For the kinetic studies of CO_2_ uptake by *M. congolense*, the versatile medium was prepared (pH 7) without any carbon sources, by omitting the addition of acetate, formate, and NaHCO_3_ (hereinafter referred to as mineral medium). CO_2_ gas injected into the headspace at the beginning of incubation was thus the sole carbon source and electron acceptor for *M. congolense* during all incubations. To minimize pH effects by high amount of CO_2_, a version of mineral medium with higher buffering capacity, 10 times higher K_2_HPO_4_ (5.0 g/L) and 5 times higher KH_2_PO_4_ (2.5 g/L) than that of the mineral medium, was employed for cultures with high CO_2_ concentration (HBC mineral medium, pH 7). *M. congolense* performed normal growth in either medium or HBC mineral medium as in the versatile medium.

### Experimental Setup

Two series of batch culture experiments were carried out for determination of CO_2_ uptake kinetics of *M. congolense*: long-term batch culture lasted for about 1 week and short-term batch culture lasted for around 3 h (see details below). For both batch cultures, *M. congolense* was cultivated in 330 mL serum bottles filled with 150 mL sterile medium and sealed with butyl rubber stoppers. Cultures were transferred several times in either mineral medium or HBC mineral medium before batch culture experiments, in order to make certain that they were adapted to the medium.

#### Long-Term Batch Culture

A series of 330 mL serum bottles filled with 150 mL mineral medium were prepared aseptically for the long-term batch culture. Firstly, the bottles were flushed thoroughly with sterile H_2_ gas (0.22 μm filtered) through butyl rubber septa and headspace pressure was kept at around 2 atmospheres eventually. Different volumes of sterile CO_2_ (0.22 μm-filter filtered), 2, 4, 8, 12, 16, 20, 24, 28, 32, 36, and 40 mL, were subsequently injected into the headspace to reach different CO_2_ partial pressure in the bottles. The mole fraction of H_2_:CO_2_ was higher than 4:1 in all bottles, so H_2_ was in excess. The bottles were incubated overnight at 37°C with continuous shaking (90 rpm) to allow the partitioning of CO_2_ between the gas and liquid phases and the dissolution of CO_2_ in water to be at equilibrium before the experiment started. Following overnight incubation, headspace pressure in each bottle was measured and gas composition was determined by gas chromatography (GC). Based on pressure and gas composition, CO_2_ partial pressure in the headspace (*p*CO_2_) were calculated and used to make a series of standard curves for describing the carbonate system in the bottles with sterile medium (details in Standard Curves).

Each of the long-term bottles was inoculated with 8 mL inoculum from an exponential growth phase culture of *M. congolense*. The bottles were incubated at 37°C and shaken at 90 rpm for about 1 week. During incubation, headspace pressure was monitored and 1 mL headspace was taken for gas composition analysis at regular intervals. 2 mL of medium was sampled aseptically once a day for optical density at 600 nm (OD600) and pH measurement.

#### Short-Term Batch Culture Experiment

*M. congolense* was incubated in either mineral medium or HBC medium with 4:1 H_2_:CO_2_ gases in the headspace. When the methanogens reached late exponential growth phase, as indicated by OD600, the short-term batch cultures were flushed thoroughly with sterile H_2_ gas (0.22 μm filtered) through a needle submerged in the liquid phase. The final pressure of H_2_ in the headspace was kept at around 2 atmospheres to ensure that H_2_ was in excess. Different volumes of sterile CO_2_ gas was subsequently injected into the headspace: 2, 4, 8, 12, 16, 20, 24, 32, and 40 mL for bottles with mineral medium and 4, 12, 14, 20, 28, 32, 40, 60, 80, and 90 mL for bottles with HBC mineral medium. Some of the above setup was repeated to check data reproducibility.

Following CO_2_ gas injection, the bottles were incubated in a rotary incubator (37°C, 90 rpm) for about 1 h to allow the partitioning of CO_2_ between the gas and mineral medium and the dissolution of CO_2_ in the medium to reach equilibrium. The headspace pressure and gas composition (CO_2_ and CH_4_) were monitored with an interval of 15–25 min for 1.5–2 h. At the beginning and end of the experiment, 2 mL liquid were removed aseptically from the bottle for OD600 and pH measurement and 2 mL for determination of cell abundance using quantitative PCR (qPCR). OD600 stayed nearly constant during the experiment. pH at the beginning of the experiment varied with CO_2_ amount injected to the bottle. The maximum pH variation was observed with 40 mL CO_2_ injected into bottles with mineral medium, where pH dropped to 6.44, compared to pH 7 before adding any CO_2_. pH dropped to 6.63 with 90 mL CO_2_ injected into HBC mineral medium bottles.

### Analytical Measurements

The pressure of headspace was monitored by gas pressure sensor during the incubation. Headspace gas composition (CO_2_ and CH_4_) was determined immediately on a gas chromatograph equipped with a thermal conductivity detector (Shimadzu-2014) and a stainless steel column packed with Poropaq Q column. The carrier gas was helium. OD600 was measured on a Genesys 10 UV-VIS spectrophotometer (ThermoFisher, USA). pH was measured using a pH meter B-71X (Horiba, Kyoto, Japan).

### Cell Abundance Estimation

Liquid samples for cell abundance were flash frozen with liquid nitrogen and stored at −20°C until analysis. DNA was extracted by using FastDNA kit (MP Biomedicals, LLC) and quantitative PCR was executed for quantification of cell abundance by using archaeal 16S rRNA primer pair–arc806F and arc915r-mod (Chen et al., 2017). Cell abundances of *M. congolense* were estimated by dividing the 16S gene copies with a factor of 3, since *M. congolense's* genome harbors three 16S rRNA gene copies (Tejerizo et al., 2017).

### Carbonate System Calculation

#### Standard Curves

Following the measurements in Long-Term Batch Culture, standard curves were generated with sterile medium for carbonate system calculation. Under gas-liquid equilibrium conditions, CO_2_ gas injected into the 330 mL bottles with 150 mL sterile mineral medium at the starting time ultimately split into two fractions, CO_2_ gas in the headspace [CO_2(g)_] and dissolved inorganic carbon (DIC) in the medium. The latter was composed of three species (dissolved CO_2_ gas–CO_2(aq)_, ${\text{HCO}}_{3}^{-}$ and ${\text{CO}}_{3}^{2-}$).

To generate standard curves, *p*CO_2_ values at equilibrium [*p*CO_2(eq)_] was firstly measured in each bottle receiving different amounts of CO_2_ (∑CO_2_). Then *p*CO_2(eq)_ was plotted against ∑CO_2_, and the resulting curve was used for the calculation of total CO_2_ in each bottle during the experiments. Furthermore, [DIC] at equilibrium [[DIC]_(eq)_] were calculated by subtracting CO_2(g)_ from ∑CO_2_, and both *p*CO_2(eq)_ and ∑CO_2_ were plotted with [DIC]_(eq)_ to generate standard curves for deduction of [DIC] during experiments.

Standard curves for carbonate system in the HBC mineral medium were generated in a similar fashion.

#### Carbonate System Calculation During Incubation

During the microbial growth, we assumed that the buffering capacity in the medium changed very little because the uptake of phosphate was minor compared to the amount of phosphate present in the medium. Thus, the carbonate system in bottles with methanogens behaved similarly as in sterile medium without microbial activity. This was supported by the fact that, when CO_2_ was nearly completely consumed by methanogens at the end of long-term batch culture experiments, medium pH returned to nearly 7, which was the initial sterile medium pH before CO_2_ injection.

Based on the above assumption, ∑CO_2_ in the bottle and [DIC] in the medium during the incubation were estimated from measured *p*CO_2_ by using the standard curves generated in Standard Curves. Concentrations of three inorganic carbon species in the medium (CO_2(aq)_, ${\text{HCO}}_{3}^{-}$ and ${\text{CO}}_{3}^{2-}$) were estimated by software CO_2_SYS (Pierrot et al., 2006) by providing *p*CO_2_ and [DIC]. In this study, carbonate is negligible because the pH range was about 6.44–7, so [DIC] ≈ [CO_2(aq)_] + [${\text{HCO}}_{3}^{-}$]. Both CO_2(aq)_ and ${\text{HCO}}_{3}^{-}$ are bioavailable carbonate species, and they are shown to be utilized by methanogens in different steps in methanogenesis and carbon fixation (Ferry, 2013). Moreover, the enzyme carbonate anhydrase, which can actively transform ${\text{HCO}}_{3}^{-}$ to CO_2_ gas or vice versa, is found to be ubiquitous in the culturable methanogens isolated so far, including *M. congolense*. Therefore, DIC is used as the main parameter for studying the CO_2_ uptake kinetics here.

### Calculation of Kinetic Parameters

#### Short-Term Batch Culture Experiment

∑CO_2_ consumption and CH_4_ production kinetics were estimated from specific ∑CO_2_ consumption rates (*V*_∑CO_2__) and specific CH_4_ production rates (*V*_CH_4__) in a range of [DIC] in the medium.

[DIC] values were estimated from the CO_2_ gas volumes injected in the bottles by fitting them to the standard curve of [DIC] and initial CO_2_ amount injected as mentioned in Standard Curves.

In each incubation, *V*_∑CO_2__ was calculated by linear fitting of the ∑CO_2_ concentration as a function of time. The ∑CO_2_ concentrations in the incubations were estimated as indicated in Standard Curves. Similarly, V_CH_4__ was estimated by linear fitting of the methane concentration in the headspace as function of time in each incubation. We assumed here that the dissolved CH_4_ was negligible due to its low solubility in water.

#### Long-Term Batch Culture

During the long-term incubation, specific ∑CO_2_ consumption rates (*V*_∑CO_2__) were calculated by dividing ∑CO_2_ consumed between two sampling points with time and normalized with cell abundance. Specific CH_4_ production rates (*V*_CH_4__) were calculated in a same manner. Since *V*_∑CO_2__ and *V*_CH_4__ changed with time during incubation, only the maximum *V*_∑CO_2__ (*r*_∑CO_2__) and *V*_CH_4__ (*r*_CH_4__) from each bottle were taken for kinetic analysis.

The growth yields (*Y*_*biomass*_) were calculated at the end of incubation when CO_2_ was nearly consumed. We assumed that the CO_2_ fraction, which was not converted to CH_4_, was assimilated into cell biomass. Thus,

$$\begin{array}{l} Y_{biomass}=1-\left[{\text{CH}}_{4\left(\text{end}\right)}\right]/\left(\left[\sum {\text{CO}}_{2\left(\text{initial}\right)}\right]-\left[\sum {\text{CO}}_{2\left(\text{end}\right)}\right]\right) \end{array}$$

Here, CH_4(end)_ refers to total CH_4_ amount produced by the end of incubation; [∑CO_2(initial)_] refers to the ∑CO_2_ amount injected at the beginning of the experiment; [∑CO_2(end)_] refers to the ∑CO_2_ amount left in the bottle at the end of the incubation.

During the exponential growth phase, cell numbers were plotted against the volume of ∑CO_2_ consumed and CH_4_ produced, respectively. The best-fit linear slopes were taken as the growth yields with respect to CO_2_ consumed and CH_4_ produced (*Y*_CO_2__ and *Y*_CH_4__).

#### Modeling of Kinetics

In summary, we estimated *V*_∑CO_2__ and *V*_CH_4__ from three different experiment setups: (1) short-term incubation with versatile mineral medium; (2) short-term incubation with HBC mineral medium; (3) long-term incubation with versatile mineral medium (*r*_∑CO_2__ and *r*_CH_4__).

We used the Michaelis-Menten equation to represent the effect of [DIC] on the performance of methanogen's ∑CO_2_ consumption rate and CH_4_ production rate, with the consideration of [DIC] threshold [DIC]^*^:

$$\begin{array}{r} {V_{\sum }}_{\text{C}{\text{O}}_{2}}=V_{\text{max}-\text{C}{\text{O}}_{2}}\times \left(\left[DIC\right]-{\left[DIC\right]}^{*}\right)/\\ \left(K_{\text{m}-\text{C}{\text{O}}_{2}}+\left[DIC\right]-{\left[DIC\right]}^{*}\right)\\ V_{\text{C}{\text{H}}_{4}}=V_{\text{max}-\text{C}{\text{H}}_{4}}\times \left(\left[DIC\right]-{\left[DIC\right]}^{*}\right)/\\ \left(K_{\text{m}-\text{C}{\text{H}}_{4}}+\left[DIC\right]-{\left[DIC\right]}^{*}\right) \end{array}$$

Where: *V*_max−CO_2__ and *V*_max−CH_4__ are the maximum specific ∑CO_2_ consumption rate and CH_4_ production rate, respectively; *K*_m−CO_2__ and *K*_m−CH_4__ are the [DIC] giving one-half the maximum specific ∑CO_2_ consumption rate and CH_4_ production rate respectively.

By the end of the batch culture experiments, headspace CO_2_ was depleted to a nearly constant partial pressure without further consumption, even though H_2_ was still in surplus. This constant CO_2_ partial pressure remained unchanged over a period even longer than the period of active CO_2_ uptake. This indicates that there is a CO_2_ and/or [DIC] threshold for *M. congolense*. The [DIC]^*^ concentration at which no active CO_2_ uptake activity could be detected was found to be 44.4 ± 0.4 μM of DIC (*N* = 7) in this study.

## Results

### Growth of *M. congolense* in Long-Term Batch Cultures

Table 1 summarizes cell growth rates, maximum ∑CO_2_ consumption (*r*_∑CO_2__) and CH_4_ (*r*_CH_4__) production rates and growth yield of *M. congolense* incubated under different initial ∑CO_2_ amount and surplus H_2_. Growth rates, *r*_CH_4__ and *r*_∑CO_2__ increased linearly with added ∑CO_2_. Growth rate increased from <0.02 d^−1^ with 2 mL CO_2_ (~0.09 mmol, [DIC] = 0.44 mM) injected to about 1.12 d^−1^ with 32 mL CO_2_ (~1.43 mmol, [DIC] = 5.13 mM) injected. Lowest *r*_CH_4__ and *r*_∑CO_2__ (0.26 and 0.21 *f* mol cell^−1^ d^−1^, respectively) were found in the bottle receiving least amount of CO_2_ (2 mL). The bottle receiving 36 mL CO_2_ showed the highest *r*_CH_4__ of about 108.99 *f* mol cell^−1^ d^−1^, while the bottle receiving 40 mL CO_2_ showed the highest *r*_∑CO_2__ of about 80.90 *f* mol^−1^ cell d^−1^. The growth yield, *Y*_biomass_-carbon assimilated for anabolism with respect to total CO_2_ assimilated, was in the range of 11–26%, but it did not show a clear trend with ∑CO_2_ amount injected. Cell specific growth yields with respect to ∑CO_2_ consumption (*Y*_CO_2__) and CH_4_ production (*Y*_CH_4__), which were estimated from data acquired during the exponential growth phase, were nearly equivalent in all conditions. *Y*_CO_2__ and *Y*_CH_4__ were consistent at around 0.8–1.8 × 10^14^ cells per mole gas consumed or produced, except for the low CO_2_ conditions (2–8 mL) where cell growth was not observed during incubation. *Y*_CO_2__ and *Y*_CH_4__ estimated in the bottles receiving <8 mL CO_2_ can be biased due to slow growth, therefore the yields were not used here.

**Table 1:** Growth parameters of *M. congolense* under different initial CO_2_ amounts injected into the bottles.

**Initial CO_**2**_ amount (mL)**	**[DIC] (mM) at equilibrium**	***p*CO_2_ at equilibrium (matm)**	**Growth rate (d^−1^)**	**Maxmium CH_4_ production rate, *r*_CH_4__ (*f*mol CH_4_ cell^−1^ d^−1^)**	**Maxmium CO_2_ consumption rate, *r*_∑CO_2__ (*f*mol CO_2_ cell^−1^ d^−1^)**	**Growth yield**		
						**Y_biomass_ (mol C per mol CO_2_)**	**Y_CO_2__ (log number of cells per mol CO_2_)**	***Y*_CH_4__ (log number of cells per mol CH_4_)**
2	0.44	2.9	<0.02	0.26	0.21	21%	N.D.	N.D.
4	0.85	6.6	0.06	3.23	2.61	16%	N.D.	N.D.
8	1.62	14.6	0.06	6.65	5.04	22%	N.D.	N.D.
12	2.33	24	0.33	20.37	18.99	17%	14.25	14.22
16	2.96	34.9	0.28	34.42	32.87	11%	13.96	13.94
20	3.55	46.9	0.56	30.72	31.09	15%	13.96	13.96
24	4.13	59	0.47	43.94	47.6	21%	13.92	13.96
28	4.68	71.8	0.84	41.03	40.33	15%	13.95	13.93
32	5.13	87.5	1.12	47.76	57.12	26%	14.09	14.19
36	5.58	102.3	0.64	108.99	69.62	24%	14.07	14.11
40	6.16	114.7	0.94	66.83	80.9	22%	13.89	13.93

In this long-term batch culture experiment, both *r*_CH_4__ and *r*_∑CO_2__ were of first order kinetics and showed a linear correlation with [DIC] instead of showing Michaelis-Menten kinetics. This implies that the saturation concentration of [DIC] for *M. congolense* is higher than 6 mM, which was the highest concentration tested in this study (Table 1). Unfortunately, attempts to further increase [DIC] by injecting more CO_2_ gas (60 mL) with the same setup led to a pH drop from 7 to <6.4 and substantial reduction of methanogenic activity (data not shown).

### Kinetics of ∑CO_2_ Consumption and CH_4_ Production

**Figure 1:**
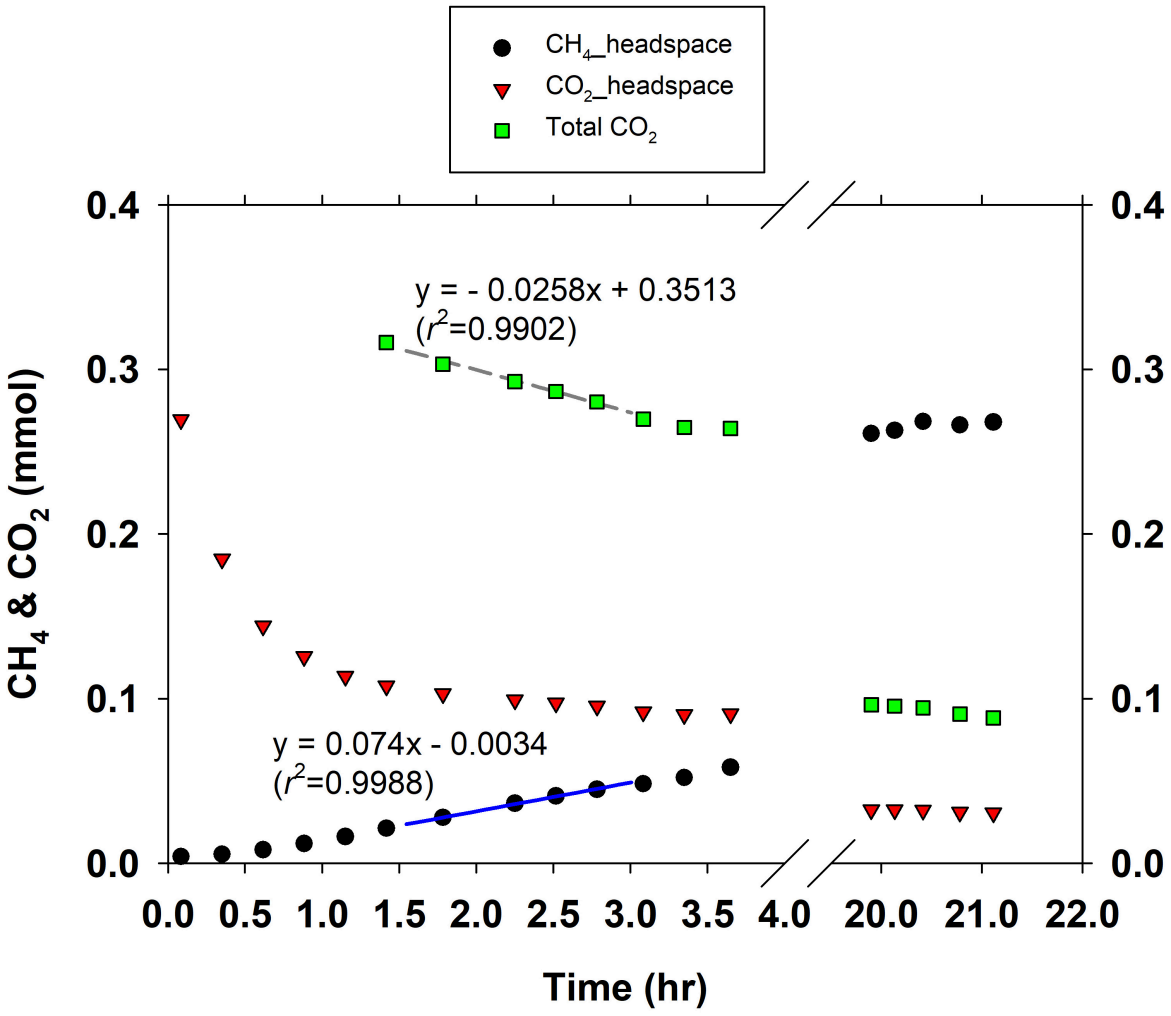
An example for determination of methane production rate and DIC consumption rate in a short-term batch culture.

Figure 1 gives an example of how *V*_∑CO_2__ and *V*_CH_4__ were estimated in short-term batch culture experiments. The CO_2_ gas in the headspace started to dissolve in the medium right after its injection, and cells started to utilize CO_2_ and produce CH_4_. CH_4_ production rates were low but kept increasing in the first 1–1.5 h during CO_2_ gas dissolution until CO_2_ equilibrium between gas and liquid phase reached. After the CO_2_ gas-liquid equilibrium, CH_4_ production rate was stable for a few hours (data not shown). To minimize the influence of increasing cell abundance due to growth during experiment, specific CH_4_ production rates were retrieved within 1–1.5 h after reaching CO_2_ gas-liquid equilibrium. In CO_2_-depleted medium *M. congolense* produced CH_4_ at extremely low rates in the presence of H_2_ (data not shown).

**Figure 2:**
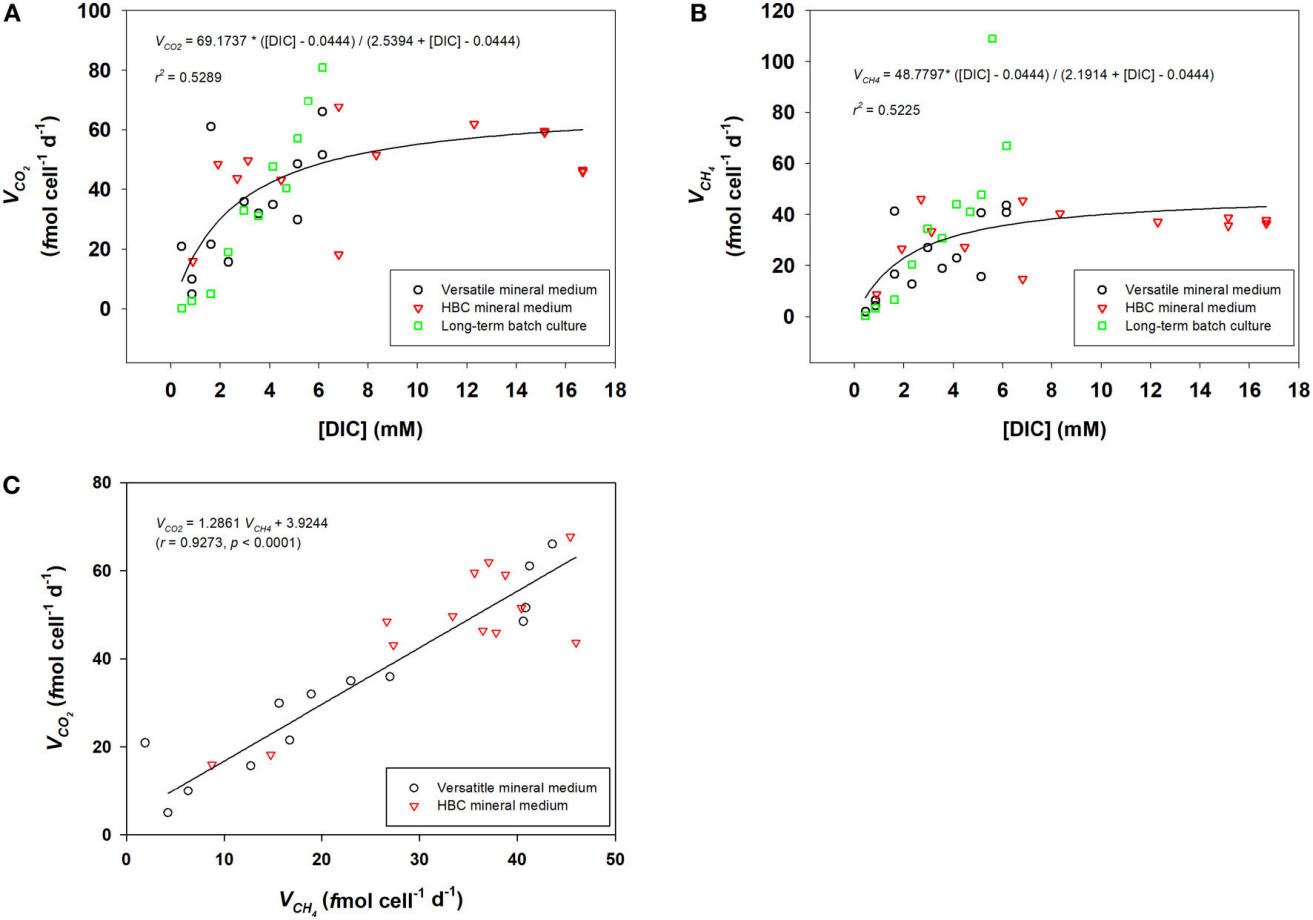
*K*_*m*_ and *V*_*m*_ of DIC consumption **(A)** and methanogenic activity **(B)** for *M. congolense*, determined from both short-term and long-term batch cultures by Michaelis-Menten fitting. **(C)**, Correlation between CO_2_ consumption rate (*V*_CO_2__) and methane production rate (*V*_CH_4__) in short-term batch cultures with both versatile mineral medium and HBC mineral medium (*N* = 25).

Figures 2A,B show the estimation of *V*_∑CO_2__ and *V*_CH_4__ from three independent experimental setups, respectively: (1) short-term incubation with versatile mineral medium; (2) short-term incubation with HBC mineral medium; (3) long-term incubation with versatile mineral medium. *V*_∑CO_2__ and *V*_CH_4__ were both dependent on available [DIC] and followed Michaelis-Menten kinetics. Maximum specific CO_2_ consumption rate (*V*_max−CO_2__) was estimated to be about 69.2 *f* mol CO_2_ cell^−1^ d^−1^ and half-saturation concentration of DIC (*K*_m−CO_2__) was about 2.5 mM. Maximum specific CH_4_ production rate of *M. congolense, V*_max−CH_4__, was about 48.8 *f* mol CH_4_ cell^−1^ d^−1^ and half-saturation concentration of DIC (*K*_m−CH_4__) was estimated to be about 2.2 mM. Reaction speeds of 80% V_max−CH_4__ could be reached at 9 mM [DIC]. Extrapolation of the fitted curve in Figure 2B shows that DIC concentrations needed for 90% V_max−CH_4__ and 100% V_max−CH_4__ would be 22 mM and 100 mM. As these values are outside the range of [DIC] that could be tested in the present setup without inferring changes in media pH, these concentrations could not be verified experimentally and should thus be interpreted with great care.

Figure 2C shows that there was good correlation between *V*_∑CO_2__ and *V*_CH_4__ for cells in late exponential growth phase in the short-term batch culture, where *V*_∑CO_2__ was about 1.3 times higher than *V*_CH_4__.

### Estimation of Methanogenic Activities Under Various CO_2_ Headspace Concentrations

**Figure 3:**
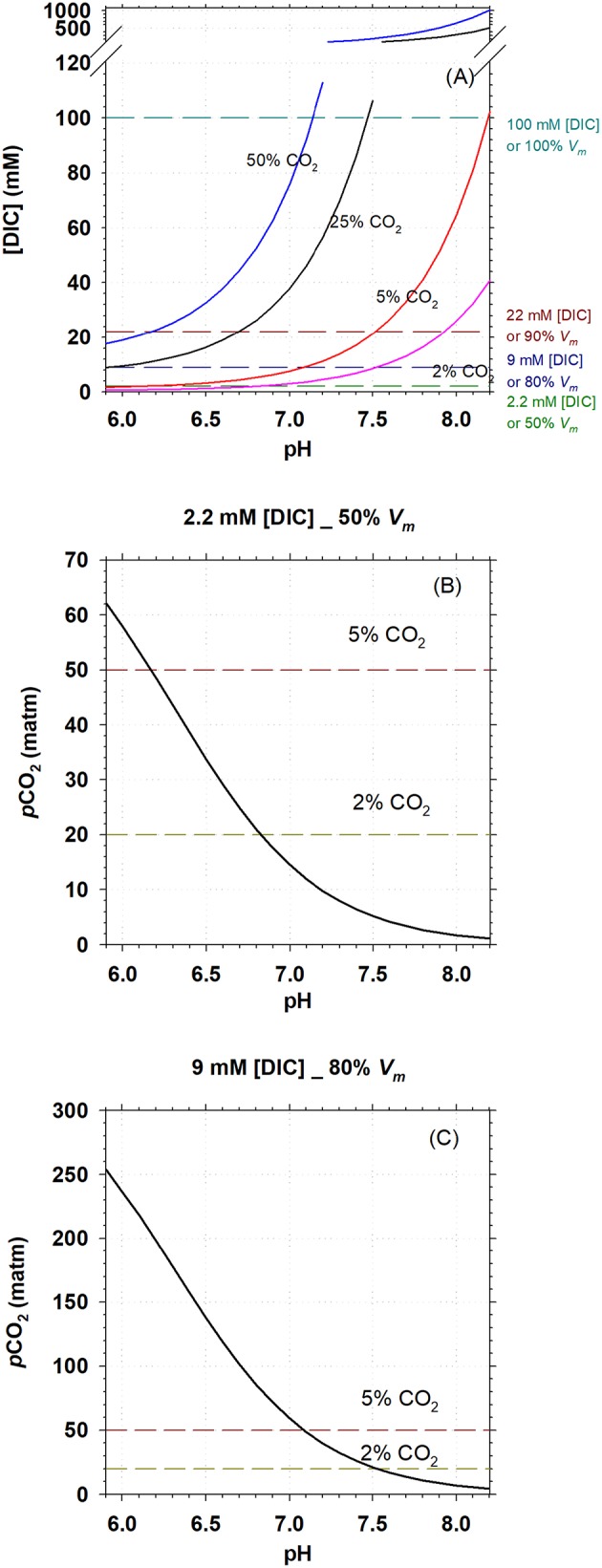
Carbonate systems, methanogenic activity, and pH in mesophilic AD. **(A)**, Calculated DIC concentration in sludge in a mesophilic AD with headspace CO_2_ concentration of 2, 5, 25, and 50%; pH was set to be within the growth range for *M. congolense*. **(B,C)**, *p*CO_2_ at pH range of 5.9–8.2 in an mesophilic AD, when [DIC] = 2.2 and 6 mM, respectively. Cell specific methanogenic rate for *M. congolense* is estimated to be 50 and 80% of *V*_*m*−*CH*_4__ at [DIC] of 2.2 and 9 mM, respectively, following the kinetic modeling from Figure 2B.

Biomethanation aims at decreasing *p*CO_2_ in the off-gas, which will directly influence [DIC] in the liquid (slurry) in AD. The [DIC] is furthermore dependent on slurry pH as this determines the CO_2_/${\text{HCO}}_{3}^{-}$ partitioning. Figure 3A shows the profile of [DIC] with pH in liquid (salinity = 0) under headspace CO_2_ concentration of 2, 5, 25, and 50%, assuming that the headspace pressure was 1 atmosphere and in equilibrium with slurry and using the growth pH range for *M. congolense* (5.9–8.2) (Cuzin et al., 2001) for calculation. It can be seen that increased *p*CO_2_ in headspace refers to higher [DIC] in the slurry and [DIC] further increases with pH. It can be calculated that [DIC] is tens of mM in the slurry in a normal mesophilic AD, where CO_2_ gas composes 25–50% of the biogas (Plugge, 2017). The lowest [DIC] is found at the lowest pH tested (5.9), about 9 mM, which can support the methanogenic rate of 80% of V_max−CH_4__.

For biomethanation, it is critical to know how much *p*CO_2_ can be lowered through upgrading CO_2_ to CH_4_ but without affecting methanogenic activity. In Figures 3B,C, we model the lowest *p*CO_2_ required to maintain 2.2 and 9 mM [DIC] at different pH so as to maintain 50 and 80% of *V*_max−CH_4__ according to the kinetic modeling. It is found that CO_2_ can be lowered to 5% and 2% at pH >6.2 and pH >6.8, respectively, with a methanogenic rate of 50% V_max−CH_4__ (Figure 3B). Correspondingly, with a methanogenic rate of 80% V_max−CH_4__, CO_2_ can be lowered to 5% and 2%, respectively, at pH >7.1 and pH >7.5 (Figure 3C).

## Discussion

Using batch-culture experiments, we provided the first estimation of CO_2_/DIC uptake kinetics of an autotrophic hydrogenotrophic methanogen, *M. congolense*. We found that the affinity for DIC was dramatically lower than that of H_2_, the other reactant involved in hydrogenotrophic methanogenesis. With a *K*_*m*_ of 2.2–2.5 mM, the affinity for DIC was shown to be a few tens to thousands times lower than the *K*_*m*_ of H_2_, 0.44–66 μM, as previously reported for other hydrogenotrophic methanogens (Kotsyurbenko et al., 2001; Karadagli and Rittmann, 2007). Likewise, DIC threshold of 44.4 μM, at which concentration the methanogenic activity could no longer be detected for *M. congolense*, was also hundreds to thousands times higher than reported H_2_ thresholds of 6–70 nM, observed for methanogens (Lin et al., 2012). Such high *K*_*m*_ and threshold of DIC might be related to the CO_2_ fixation pathway used by methanogens. *M. congolense* utilizes the Wood-Ljungdahl pathway for CO_2_ fixation, which has previously been shown to have the highest *K*_*m*_ of DIC among the six autotrophic inorganic carbon assimilation pathways (Raven et al., 2012). From an evolutionary perspective, poor affinity for DIC is in accordance with the ubiquitous distribution of methanogens in habitats with high CO_2_ concentrations, such as anaerobic digesters, animal guts and sediments. Maximum CH_4_ production rate (*V*_max−CH_4__, 48.8 *f* mol CH_4_ cell^−1^ d^−1^) of *M. congolense*, estimated from the kinetic model here is comparable to methanogenic rates found for methanogens from other complex environments incubated at a comparable temperature (35°C): 108-135 *f* mol CH_4_ cell^−1^ d^−1^ in anaerobic reactors (Li and Noike, 1992) and 31.5 *f* mol CH_4_ cell^−1^ d^−1^ in lake sediments (Lay et al., 1996).

Addition of different amounts of CO_2_ gas at the beginning of the batch culture experiments caused the decrease of media pH due to the dissolution of CO_2_ gas into the medium, where pH decreased more in bottles receiving higher amounts of CO_2_. A further challenge was that the CO_2_ concentration changed constantly during the incubation, due to continuous CO_2_ consumption. Therefore, we controlled the amount of CO_2_ added to keep pH within an optimal range for *M. congolense* (pH 5.9–8.2) (Cuzin et al., 2001), so that growth and methanogenic activity of *M. congolense* were not affected during our trials. A previous study on an obligate hydrogenotrophic autotrophic methanogen, *Methanocaldococcus* strain JH146, showed that pH did not affect methanogenic activity when it was within the range for optimal growth (Eecke et al., 2013). In our experiments, addition of 2–40 mL CO_2_ lowered pH from 7 to 6.44–6.94 in the mineral medium, which has lower buffering capacity but methanogenic rates kept increasing with CO_2_ amount and reached a rate of ~44 *f* mol CH_4_ cell^−1^ d^−1^ with 40 mL CO_2_ (pH = 6.44). Moreover, specific ∑CO_2_ consumption rates and CH_4_ production rates determined from all three different experimental setups here were in good agreement with respect to [DIC], regardless of buffering capacity or incubation time (Figure 2). Thus, pH seems to have little impact on the rates within the experimental range. However, addition of excessive CO_2_ beyond the buffering capacity was shown to greatly inhibit methanogenic activity: addition of 60 mL CO_2_ into the mineral medium decreased pH to 6.01 and resulted in a dramatic reduction of methanogenic activity to ~12 *f* mol CH_4_ cell^−1^ d^−1^.

Our study also shows that DIC concentration influences the microbial growth rate. This is revealed by a clear reduction of growth rate from 1.12 d^−1^ at 5.13 mM [DIC] to <0.02 d^−1^ at the lowest DIC concentrations tested (0.44 mM) in the long-term batch culture experiment (Table 1). A previous study showed that the mixotrophic methanogen, *Methanosarcina barkeri*, has very slow growth and low methanogenic rate when incubated with only H_2_ but lacking CO_2_ (Weimer and Zeikus, 1978).

Here we showed that growth of *M. congolense* was limited when [DIC] was lower than 1.6 mM, although methanogenesis still continued at low rates. Methanogens fix CO_2_ autotrophically into biomass through the Wood-Ljungdahl pathway, with which its methanogenesis pathway is associated (Berg, 2011). Therefore, whether the lowered growth rate at low [DIC] was due to reduced assimilation of carbon for biomass formation, or due to a reduced energy generation from methanogenesis remains unknown. Nonetheless, cell growth yield at the end of long-term incubation seems to be consistent for all DIC concentration tested: the methanogen converts approximately four moles of CO_2_ into CH_4_ for each mole of CO_2_ incorporated into biomass (Table 1). Similar fraction of CO_2_ was assimilated into biomass by late exponential growth phase cells during our short-term batch incubation, as ∑CO_2_ consumption rate was about 1.3 times higher than methane production rate for all bottles (Figure 2C). The ratio of CO_2_ used in dissimilatory and assimilatory metabolisms were thus independent of the [DIC] concentration.

As *p*CO_2_ concentrations in anaerobic digesters is often high (25–50%), our data showed that inorganic carbon availability might not limit methanogenic activity of *M. congolense* under standard operating conditions of an anaerobic digester. Fermentation processes in the sludge will furthermore supply CO_2_ to the hydrogenotrophic methanogens and hereby decrease the likelihood of CO_2_ limitation under standard conditions. This study is of primary importance for biomethanation, a power-to-gas technology used for biogas upgrading to increase CH_4_ concentration in the produced biogas through reduction of the CO_2_ concentration. Knowledge on methanogen's CO_2_/DIC kinetics is relevant as low CO_2_ concentrations (<2%) are required to fulfill criteria for injection of upgraded biogas to the natural gas grid. If *M. congolense* is treated as a representative of hydrogenotrophic methanogens, the biogas can be upgraded to >98% CH_4_ (<2% CO_2_) at 80% V_max−CH_4__ when slurry pH in the reactor is >7.5 (Figure 3C) and 50% V_max−CH_4__ at pH >6.8 (Figure 3B). Our result suggests that the availability of bioavailable inorganic carbon under low CO_2_ concentration might not greatly decrease methanogenic activity during biogas upgrading, but other factors, such as pH, might have greater impact on methanogenic rates.

A decrease in methanogenic activities to 50% of the maximum was shown in previous studies at different CO_2_ concentrations: 2.9% CO_2_ (59 mM [DIC] in methanogenic manure samples, Garcia-Robledo et al., 2016) and 10% CO_2_ (257 mM [DIC] in anaerobic digestate, Agneessens et al., 2017). These [DIC] concentrations were higher than the *K*_*m*_ values reported here for *M. congolense*, which would indicate that the organisms in these studies either had lower affinities for [DIC]/CO_2_ than *M. congolense*, or that their methanogenic activities were inhibited by other factors like NH_3_ or pH. The pH levels of 8.2 (Garcia-Robledo et al., 2016) and 8.3 (Agneessens et al., 2017), were close to the value of 8.5 reported to be inhibitory to the biomethanation process (Angelidaki et al., 2018). As bicarbonate is the dominant buffering system in anaerobic slurries, it is often difficult to separate effects by high pH from effects by low CO_2_ concentrations here, as these are inversely related. Through the pure culture study on *M. congolense* reported here, it was possible to separate the direct pH effect from low concentrations of CO_2_ and hereby elucidate microbial physiological limitations to the process of biomethanation of a methanogenic type strain. The results are applicable to both *in situ* methanation, where H_2_ and CO_2_ are converted by methanogens in the main reactor, and separate *ex situ* reactor harboring specialized methanogenic communities.

## Conclusions

Although CO_2_ affinity of *M. congolense* is many times higher than H_2_ affinity, CO_2_ concentrations will only become severely limiting for biomethanation at very low [DIC] concentrations. Experiments were only carried out on a single methanogenic strain here and further testing of other methanogens will reveal if they elicit a similar affinity for CO_2_.

## Author Contributions

XC performed the experiments and drafted the manuscript. All authors designed the study, interpreted the data and wrote the manuscript.

### Conflict of Interest Statement

The authors declare that the research was conducted in the absence of any commercial or financial relationships that could be construed as a potential conflict of interest.
